# diceR: an R package for class discovery using an ensemble driven approach

**DOI:** 10.1186/s12859-017-1996-y

**Published:** 2018-01-15

**Authors:** Derek S. Chiu, Aline Talhouk

**Affiliations:** 1 Department of Molecular Oncology, BC Cancer Agency, Vancouver, BC Canada; 2 Department of Pathology and Laboratory Medicine, University of British Columbia, Vancouver, BC Canada

**Keywords:** Data mining, Cluster analysis, Ensemble, Consensus, Cancer

## Abstract

**Background:**

Given a set of features, researchers are often interested in partitioning objects into homogeneous clusters. In health research, cancer research in particular, high-throughput data is collected with the aim of segmenting patients into sub-populations to aid in disease diagnosis, prognosis or response to therapy. Cluster analysis, a class of unsupervised learning techniques, is often used for class discovery. Cluster analysis suffers from some limitations, including the need to select up-front the algorithm to be used as well as the number of clusters to generate, in addition, there may exist several groupings consistent with the data, making it very difficult to validate a final solution. Ensemble clustering is a technique used to mitigate these limitations and facilitate the generalization and reproducibility of findings in new cohorts of patients.

**Results:**

We introduce *diceR (diverse cluster ensemble in R)*, a software package available on CRAN: https://CRAN.R-project.org/package=diceR

**Conclusions:**

*diceR* is designed to provide a set of tools to guide researchers through a general cluster analysis process that relies on minimizing subjective decision-making. Although developed in a biological context, the tools in *diceR* are data-agnostic and thus can be applied in different contexts.

**Electronic supplementary material:**

The online version of this article (doi: 10.1186/s12859-017-1996-y) contains supplementary material, which is available to authorized users.

## Background

Cluster analysis has been used in cancer research to discover new classifications of disease and improve the understanding of underlying biological mechanisms. This technique belongs to a set of unsupervised statistical learning methods used to partition objects and/or features into homogeneous groups or clusters [1]. It provides insight, for example, to how co-regulated genes associate with groupings of similar patients based on features of their disease, such as prognostic risk or propensity to respond to therapy. Many clustering algorithms are available, though none stand out as universally better than the others. Different algorithms may be better suited for specific types of data, and in high dimensions it is difficult to evaluate whether algorithm assumptions are met. Furthermore, researchers must set the number of clusters a priori for most algorithms. Additionally, several clustering solutions consistent with the data are possible, making the ascertainment of a final result without considerable reliance on additional extrinsic information difficult [2]. Many internal clustering criteria have been proposed to evaluate the output of cluster analysis. These generally consist of measures of compactness (how similar are objects within the same cluster), separation (how distinct are objects from different clusters), and robustness (how reproducible are the clusters in other datasets) [2–4]. External evaluation can also be used to assess how resulting clusters and groupings corroborate known biological features. Researchers may choose to use internal clustering criteria only for performance evaluation [5, 6] to keep the analysis congruent with an unsupervised approach.

Ensemble methods are a popular class of algorithms that have been used in both the supervised [7, 8] and unsupervised learning setting. In the unsupervised setting, cluster ensembles have been proposed as a class of algorithms that can help mitigate many of the limitations of traditional cluster analysis by combining clustering results from multiple “experts” [2, 9]. Ensembles are achieved by generating different clusterings, using different subsets of the data, different algorithms, or different number of clusters, and combining the results into a single consensus solution. Ensemble methods have been shown to result in a more robust clustering that converges to a true solution (if a unique one exists) as the number of experts is increased [9–11]. The agnostic approach of ensemble learning makes the technique useful in many health applications, and non-health applications such as clustering communities in social network analysis (Maglaras et al., 2016) and classifying credit scores (Koutanaei et al., 2015).

## Implementation

In this paper, we introduce diverse cluster ensemble in R *(diceR*), a software package built in the R statistical language (version 3.2.0+) that provides a suite of functions and tools to implement a systematic framework for cluster discovery using ensemble clustering. This framework guides the user through the steps of generating diverse clusterings, ensemble formation, and algorithm selection to the arrival at a final consensus solution, most consistent with the data. We developed a visual and analytical validation framework, thereby integrating the assessment of the final result into the process. Problems with scalability to large datasets were solved by rewriting some of the functions to run parallel on a computing cluster. *diceR* is available on CRAN.

## Results and discussion

**Fig. 1:**
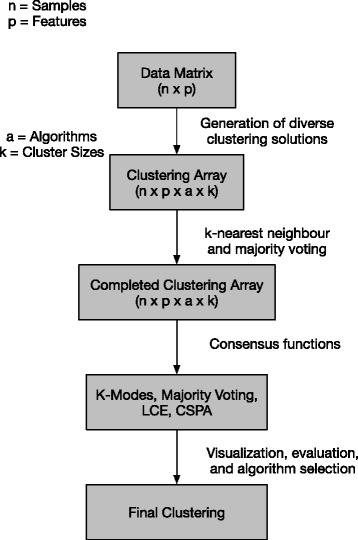
Ensemble clustering pipeline implemented in diceR. The analytical process is carried out by the main function of the package: dice

The steps performed in the *diceR* framework are summarized below and in Fig. 1; a more detailed example can be found in the Additional file 1 and at https://alinetalhouk.github.io/diceR

### Diverse cluster generation

The full process is incorporated into a single function dice that wraps the different components described herein. The input data consists of a data frame with rows as samples and columns as features. Cluster generation is obtained by applying a variety of clustering algorithms (e.g. k-means, spectral clustering, etc.), distance metrics (e.g. Euclidean, Manhattan, etc.), and cluster sizes to the input data (please consult the supplementary methods for the list of algorithms and clustering distances currently implemented). In addition to algorithms and distances implemented within *diceR*, a simple framework is available for the user to input the algorithm or distance of their choosing. Every algorithm is applied to several subsets of the data, each consisting of 80% of the original observations. As a result of subsampling, not every sample is included in each clustering; the data is “completed” using k-nearest neighbor and majority voting.
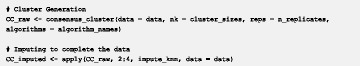


The output of the cluster generation step is an array of clustering assignments computed across cluster sizes, algorithms, and subsamples of the data (See “Clustering Array” and “Completed Clustering Array” in Fig. 1). This technique extends the consensus clustering method proposed by Monti et al. [12] to include a consensus across algorithms.

### Consensus ensemble

A cluster ensemble is generated by combining results from the cluster generation step. *diceR* implements four methods for consensus formation: Majority Voting [13], K-modes [14], Link-Based Cluster Ensembles (LCE) [10], and Cluster-based Similarity Partitioning Algorithm (CSPA) [9, 15] (See Fig. 1). Thus, the final ensemble is a consensus across samples *and* algorithms.



There is also an option to choose a consensus cluster size using the proportion of ambiguous clustering (PAC) metric [4]. The cluster size corresponding to the smallest PAC value is selected, since low values of PAC indicate greater clustering stability. Additionally, the user can allocate different weights to the algorithms in the ensemble, proportional to their internal evaluation index scores.

### Visualization and evaluation

For each clustering algorithm used, we calculate internal and external validity indices [5, 6]. *diceR* has visualization plots to compare clustering results between different cluster sizes. The user can monitor the consensus cumulative distribution functions (CDFs), relative change in area under the curve for CDFs, heatmaps, and track how cluster assignments change in relation to the requested cluster size.
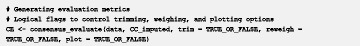


**Fig. 2:**
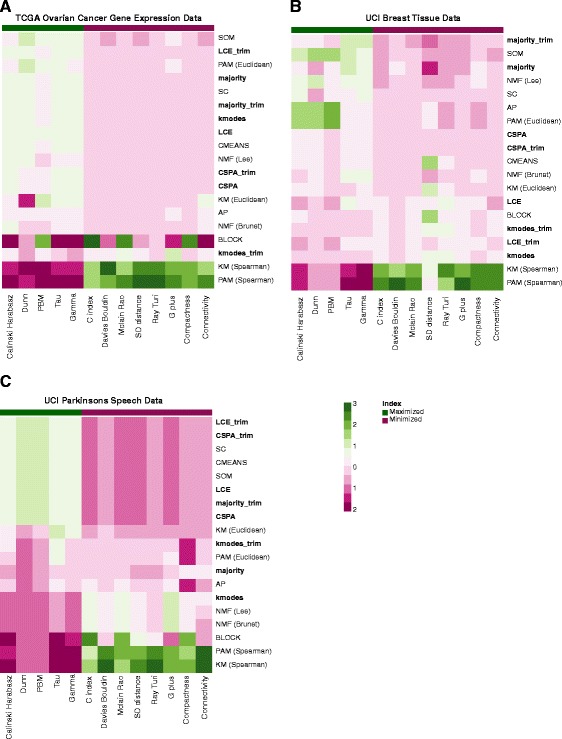
A comparative evaluation using diceR applied to three datasets. Using 10 clustering algorithms, we repeated the clustering of each data set, each time using only 80% of the data. Four ensemble approaches were considered. The ensembles were constructed using all the individual clusterings and were repeated by omitting the least performing algorithms (the trim version in the figure). Thirteen internal validity indices were used to rank order these algorithms based on performance from top to bottom. Indices were standardized so their performance is relative to each other. The green/red annotation tracks at the top indicate which indices should be maximized or minimized respectively. Ensemble methods were highlighted using a bold font

A hypothesis testing mechanism based on the SigClust method is also implemented in *diceR* to assess whether clustering results are statistically significant [16]. This allows quantification of the confidence in the partitions. For example, we can test whether the number of statistically distinct clusters is equal to two or three, as opposed to just one (i.e. unimodal distribution no clusters). In Fig. 2 we present a visualization of the results of a comparative analysis.

### Algorithm selection

Poor-performing algorithms can affect a cluster ensemble’s performance, so one way to limit that is to include only the top N performing algorithms in the ensemble [17]. To this end, the internal validity indices for all algorithms are computed (see Additional file 1 for full list of indices). Then, rank aggregation is used to select a subset of algorithms that perform well across all indices [18]. The resulting subset of algorithms is selected for inclusion in the cluster ensemble. Our “diverse” strategy is not to impose diversity onto the ensemble, but to *consider* a diverse set of algorithms and ultimately allow the data to select which best performing algorithms to retain. This step of the analysis continues to be an active area of research and is subject to revision and improvements.

## Conclusions

The software we have developed provides an easy-to-use interface for researchers of all fields to use for their cluster analysis needs. More clustering algorithms will be added to *diceR* as they become available.

## Additional file

Additional file 1: A detailed tutorial and example of Cluster Analysis using diceR. (PDF 326 kb)

## Abbreviations

CDF: Cumulative distribution function
CSPA: Cluster-Based Partitioning Algorithm
diceR: Diverse cluster ensemble in R
LCE: Linkage-Based Cluster Ensembles
PAC: Proportion of ambiguous clustering
