# Supplementary material for: diceR: an R package for class discovery using an ensemble driven approach
Source: BMC Bioinformatics. 2018 Jan 15;19:11. doi: 10.1186/s12859-017-1996-y (PMC5769335; doi:10.1186/s12859-017-1996-y)
Supplement: Additional file 1: — A detailed tutorial and example of Cluster Analysis using diceR. (PDF 326 kb) [file 12859_2017_1996_MOESM1_ESM.pdf]

Cluster Analysis using diceR

Introduction

Cluster analysis is a way of "slicing and dicing" data to allow the grouping together of similar entities and the separation of dissimilar ones. Issues arise due to the existence of a diverse number of clustering algorithms, each with different techniques and inputs, and with no universally optimal methodology. Thus, a framework for cluster analysis and validation methods are needed. Our approach is to use cluster ensembles from a diverse set of algorithms so that the final class labels. This ensures that the data has been considered from several angles and using a variety of methods. We have currently implemented about 15 clustering algorithms, and we provide a simple framework to add additional algorithms (see `example("consensus_cluster")`). Although results are dependent on the subset of algorithms chosen for the ensemble, the intent is that we capture a variety of clustering and select those that are most consistent with the data.

You can install `diceR` from CRAN with:

```
install.packages("diceR")
```

Or get the latest development version from GitHub:

```
# install.packages("devtools")
devtools::install_github("AlineTalhouk/diceR")
```

```
library(diceR)
library(dplyr)
library(ggplot2)
library(pander)
data(hgsc)
hgsc <- hgsc[1:100, 1:50]
```

We load an excerpt of an expression data set from TCGA of 100 high grade serous carcinoma samples measured on 50 genes.

Clustering Algorithms

The list of clustering algorithms available for use are:

- `"nmf"`: Nonnegative Matrix Factorization (using Kullback-Leibler Divergence or Euclidean distance)
- `"hc"`: Hierarchical Clustering
- `"diana"`: DIvisive ANAlysis Clustering
- `"km"`: K-Means Clustering
- `"pam"`: Partition Around Medoids
- `"ap"`: Affinity Propagation
- `"sc"`: Spectral Clustering using Radial-Basis kernel function
- `"gmm"`: Gaussian Mixture Model using Bayesian Information Criterion on EM algorithm
- `"block"`: Biclustering using a latent block model
- `"som"`: Self-Organizing Map (SOM) with Hierarchical Clustering
- `"cmeans"`: Fuzzy C-Means Clustering
- `"hdbscan"`: Hierarchical Density-based Spatial Clustering of Applications with Noise (HDBSCAN)

They are passed as a character vector into the `algorithms` parameter of `consensus_cluster()`. Note that the list continues to evolve as we implement more algorithms into `diceR`.

Cluster Models

The clustering algorithms can be categorized under different models or paradigms based on how the clusters are formed. We provide a brief overview to guide the initial selection of algorithms since no single algorithm works for every data model.[1]

- Connectivity Models: `"hc"` and `"diana"` are hierarchical models based on connecting objects using a particular similarity or distance metric.
- Centroid Models: `"km"` and `"pam"` uses initial cluster centers calculated based on the mean of the objects, where `"pam"` restricts the centroid to be an actual object. These models are good for modeling data that produces spherical clusters. `"ap"` is similar to `"pam"` because it calculates "exemplars" (like centroids) of the data but is advantageous because initialization of k (number of clusters) is not required.[2] `"cmeans"` is similar to `"km"` except that points can be assigned to more than one cluster.
- Distribution Models: `"gmm"` assumes a Gaussian mixture model used by the EM algorithm for clustering.[3] The gain in model complexity is traded with utility since the assumption is quite strong.
- Density Models: `"hdbscan"` runs hierarchical clustering on the DBSCAN result.[4,5] These models assume that each cluster has high density, so when there are overlapping regions, the algortihms may not be able to separate objects that well.
- Subspace Models: `"block"` clusters both samples and features, so is useful when you want to associate these pairs of cluster entities (e.g. are there functional gene groups that associate with sample subtypes?). Our implementation uses the latent block model.[6]
- Neural Models: our implementation of `"som"` reduces the data to a two-dimensional subspace using a neural network model, before performing hierarchical clustering.[7] A lot of the noise is phased out and cluster stability can be improved from using `"hc"` alone.
- Spectral Clustering: Similar to `"som"`, `"sc"` also performs dimensionality reduction before clustering, but instead uses eigenvectors of a kernel matrix.[8] We use a radial-basis kernel function in the package.
- Non-negative Matrix Factorization: the computational complexity with `"nmf"` is doubled compared to other algorithms because data manipulations need to performed to transform the input data into a non-negative matrix. Otherwise, NMF has a natural clustering result that can be used to group together samples and/or features.[9]

Distance Measures

The list of distance measures available for use are those found in `stats::dist` and the spearman distance:

- `"euclidean"`: see `stats::dist`
- `"maximum"`: see `stats::dist`
- `"manhattan"`: see `stats::dist`
- `"canberra"`: see `stats::dist`
- `"binary"`: see `stats::dist`
- `"minkowski"`: see `stats::dist`
- `"spearman"`: based on `bioDist::spearman.dist`

They are passed as a character vector into the `distance` parameter of `consensus_cluster()`. Only the `"hc"`, `"diana"`, `"km"`, `"pam"` algorithms use the `distance` parameter.

Validity Indices

Internal

The list of internal validity indices used for evaluating clusters are:

- "C_index", "Calinski_Harabasz", "Davies_Bouldin", "Dunn", "McClain_Rao", "PBM", "SD_Dis", "Ray_Turi", "Tau", "Gamma", "G_plus", "Silhouette", "S_Dbw" from `clusterCrit::intCriteria`

- "Compactness" from the `LinkCluE` package
- "Connectivity" from `clValid::connectivity`

External

The list of external validity indices used for evaluating clusters are:

- "Hubert", "Jaccard", "McNemar", "Precision", "Rand", "Recall" from `clusterCrit::extCriteria`
- "NMI" calculated using `infotheo::entropy`
- Overall confusion matrix statistics from `caret::confusionMatrix`: "Overall Accuracy", "Cohen's kappa", "No Information Rate", "P-Value [Acc > NIR]"
- Averaged class-specific confusion matrix statistics: "Sensitivity", "Specificity", "PPV", "NPV", "Detection Rate", "Accuracy", "Balanced Accuracy"

Consensus Clustering

When Monti et al. (2003) first introduced consensus clustering, the idea was to use one clustering algorithm on bootstrapped subsamples of the data. We implement some extensions where a consensus is reached across subsamples *and* across algorithms. The final cluster assignment is then computed using statistical transformations on the ensemble cluster.

The base function of this package is `consensus_cluster()`, which outputs cluster assignments across subsamples and algorithms, for different k (number of clusters). For example, let's say we were interested in clustering the `hgsc` data into 3 or 4 clusters, using 80% resampling on 5 replicates, for these clustering algorithms: Hierarchical Clustering, PAM, and DIvisive ANAlysis Clustering (DIANA). Euclidean distance is used for all algorithms.

```
CC <- consensus_cluster(hgsc, nk = 3:4, p.item = 0.8, reps = 5,
                        algorithms = c("hc", "pam", "diana"))
```

The output is a 4-dimensional array: rows are samples, columns are different bootstrap subsample replicates, slices are algorithms, and each "box" (4th dimension) is for a different k. Below are the first few cluster assignments for each replicate in the DIANA algorithm for k = 3.

```
co <- capture.output(str(CC))
strwrap(co, width = 80)
#> [1] "int [1:100, 1:5, 1:3, 1:2] 1 3 1 3 1 1 1 NA 1 1 ..."
#> [2] "- attr(*, \"dimnames\")=List of 4"
#> [3] "..$ : chr [1:100] \"TCGA.04.1331_PRO.C5\" \"TCGA.04.1332_MES.C1\""
#> [4] "\"TCGA.04.1336_DIF.C4\" \"TCGA.04.1337_MES.C1\" ..."
#> [5] "..$ : chr [1:5] \"R1\" \"R2\" \"R3\" \"R4\" ..."
#> [6] "..$ : chr [1:3] \"HC_Euclidean\" \"PAM_Euclidean\" \"DIANA_Euclidean\""
#> [7] "..$ : chr [1:2] \"3\" \"4\""
```

Table 1: Cluster Assignments for DIANA, k = 3

	R1	R2	R3	R4	R5
**TCGA.04.1331_PRO.C5**	1	2	NA	NA	NA
**TCGA.04.1332_MES.C1**	1	2	1	1	NA
**TCGA.04.1336_DIF.C4**	2	NA	NA	1	NA
**TCGA.04.1337_MES.C1**	2	1	3	1	2
**TCGA.04.1338_MES.C1**	2	1	3	1	2
**TCGA.04.1341_PRO.C5**	1	2	1	2	1

Note the unavoidable presence of NAs because we used 80% subsampling. This can be problematic in certain downstream ensemble methods, so we can impute missing values using K-Nearest Neighbours beforehand. There might still be NAs after KNN because of how the decision threshold was set. The remaining missing values can be imputed using majority voting.

```
CC <- apply(CC, 2:4, impute_knn, data = hgsc, seed = 1)
CC_imputed <- impute_missing(CC, hgsc, nk = 4)
sum(is.na(CC))
#> [1] 5
sum(is.na(CC_imputed))
#> [1] 0
```

We can carry on the analysis using either CC or CC_imputed.

Consensus Functions

diceR provides functions for retrieving useful summaries and other results for consensus clustering.

- consensus_matrix()
- consensus_combine()
- consensus_evaluate()

Compute consensus matrix with consensus_matrix()

The consensus matrix is an n by n matrix, where n is the number of samples. Each element is a real-valued number between 0 and 1 inclusive, representing the proportion of times that two samples were clustered together out of the times that the same samples were chosen in the bootstrap resampling. The diagonal are all one's. Suppose we wanted to compute the consensus matrix for PAM, k = 4, and visualize using graph_heatmap():

```
pam.4 <- CC[, , "PAM_Euclidean", "4", drop = FALSE]
cm <- consensus_matrix(pam.4)
dim(cm)
#> [1] 100 100
```

```
hm <- graph_heatmap(pam.4)
```

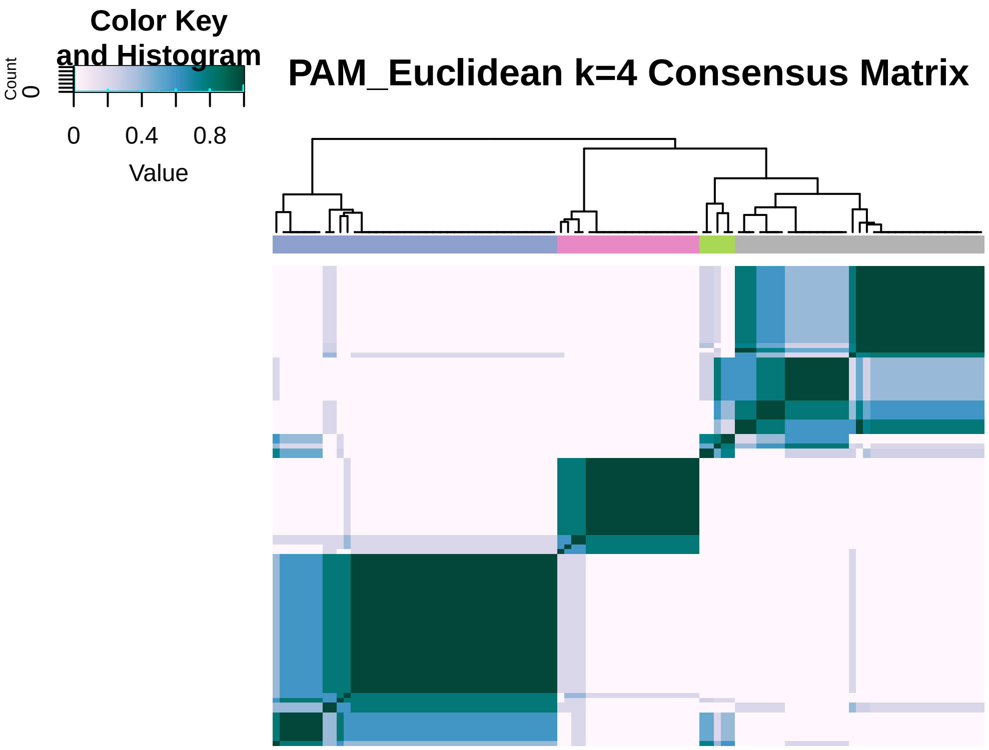

Combine consensus summaries with `consensus_combine()`

If we wish to separately extract consensus matrices and consensus classes for every algorithm, `consensus_combine()` is a convenient wrapper to do so. Setting `element = "matrix"` returns a list of consensus matrices. On the other hand, setting `element = "class"` returns a matrix with rows as samples, and columns as clustering assignments for each algorithm.

```
ccomb_matrix <- consensus_combine(CC, element = "matrix")
ccomb_class <- consensus_combine(CC, element = "class")
```

```
str(ccomb_matrix, max.level = 2)
#> List of 2
#>  $ 3:List of 3
#>   ..$ HC_Euclidean   : num [1:100, 1:100] 1 0.4 1 0.4 1 1 1 1 1 0.8 ...
#>   ..$ PAM_Euclidean  : num [1:100, 1:100] 1 1 0.2 1 0 1 1 0.8 0 0 ...
#>   ..$ DIANA_Euclidean: num [1:100, 1:100] 1 0.6 0 0 0 1 1 0 0 1 ...
#>  $ 4:List of 3
#>   ..$ HC_Euclidean   : num [1:100, 1:100] 1 0.2 1 0 1 1 1 1 1 0.8 ...
#>   ..$ PAM_Euclidean  : num [1:100, 1:100] 1 0.8 0 0.4 0 1 1 0.8 0 0 ...
#>   ..$ DIANA_Euclidean: num [1:100, 1:100] 1 0.6 0 0 0 1 1 0 0 1 ...
```

Table 2: Consensus Classes

HC_Euclidean	PAM_Euclidean	DIANA_Euclidean
1	1	1
2	1	1
1	2	2
3	1	2
1	2	2
1	1	1

HC_Euclidean	PAM_Euclidean	DIANA_Euclidean

One can feed in `ccomb_class` (instead of `CC`) into `consensus_matrix()` to obtain a consensus matrix across both subsamples **and** algorithms.

A situation might also arise where we initially decided on using 3 clustering algorithms for the ensemble, but later wish to add additional algorithms for analysis. `consensus_combine()` takes in any number of ensemble objects (e.g. `CC` and `CC2`) and combines the results.

```
CC2 <- consensus_cluster(hgsc, nk = 3:4, p.item = 0.8, reps = 5,
                         algorithms = "km")
ccomb_class2 <- consensus_combine(CC, CC2, element = "class")
```

Table 3: Consensus Classes with KM added

HC_Euclidean	PAM_Euclidean	DIANA_Euclidean	KM_Euclidean
1	1	1	1
2	1	1	1
1	2	2	2
3	1	2	3
1	2	2	2
1	1	1	1

Evaluate, trim, and reweigh algortihms with `consensus_evaluate()`

Internal cluster validation indices assess the performance of results by taking into account the compactness and separability of the clusters. We choose a variety of indices on which to compare the collection of clustering algorithms. We use the PAC (Proportion of Ambiguous Clusters), the proportion of entries in a consensus matrix that are strictly between `lower` (defaults to 0) and `upper` (defaults to 1), to give a measure of cluster stability. In addition, if no reference class is provided, we calculate the average PAC across algorithms within each `k`, and choose the `k` with the greatest average PAC. If there *is* a reference class, `k` is the number of distinct classes in the reference.

```
ccomp <- consensus_evaluate(hgsc, CC, CC2, plot = FALSE)
```

Table 4: Internal Indices for k = 4 (continued below)

Algorithms	c_index	calinski_harabasz	davies_bouldin	dunn	mcclain_rao	pbm
HC_Euclidean	0.2092	3.949	0.5132	0.4524	0.7278	13.25
PAM_Euclidean	0.1796	15.09	2.056	0.2979	0.782	8.456
DIANA_Euclidean	0.1909	12.51	1.507	0.2663	0.7848	5.698
KM_Euclidean	0.1195	15.84	1.912	0.2541	0.7398	8.839

sd_dis	ray_turi	tau	gamma	g_plus	silhouette	s_dbw	Compactness	Connectivity
0.2666	0.5899	0.3188	0.7036	0.03044	NA	NA	7.783	15.93
0.4754	1.225	0.3756	0.5701	0.09328	0.08144	NA	6.875	72.87
0.4673	1.685	0.3863	0.5516	0.11	0.02748	NA	7.071	50.82
0.4449	1.142	0.4683	0.7021	0.06624	0.06399	NA	6.84	73.99

We see that the biclustering algorithm is the least ambiguous and also most well-clustered (high compactness and separability).

Some algorithms perform too poorly to deserve membership in the cluster ensemble. We consider the relative ranks of each algorithm across all internal indices, and compute their sum. All algorithms below a certain quantile for the sum rank are trimmed (removed). By default this quantile is 75%.

After trimming, we can optionally choose to reweigh the algorithms based on the internal index magnitudes. Of course, we take into account the direction of optimality (higher is better is lower is better). Algorithms reweighed are then fed into the consensus functions. This is done by replicating each algorithm by a scalar factor that is proportional to its weight. For example, if we have two algorithms A and B, and A is given a weight of 80% and B is given a weight of 20%, then we make 4 copies of A and 1 copy of B. To minimize computational time, the total number of copies out of all algorithms has an upper bound of 100. Without reweighing, each algorithm is given equal weight.

```
ctrim <- consensus_evaluate(hgsc, CC, CC2, trim = TRUE, reweigh = FALSE, n = 2)
```

```
str(ctrim, max.level = 2)
#> List of 5
#>  $ k       : int 3
#>  $ pac     :'data.frame':    2 obs. of  5 variables:
#>   ..$ k              : chr [1:2] "3" "4"
#>   ..$ HC_Euclidean   : num [1:2] 0.487 0.432
#>   ..$ PAM_Euclidean  : num [1:2] 0.248 0.251
#>   ..$ DIANA_Euclidean: num [1:2] 0.215 0.242
#>   ..$ KM_Euclidean   : num [1:2] 0.275 0.242
#>  $ internal:List of 2
#>   ..$ 3:'data.frame':    4 obs. of  16 variables:
#>   ..$ 4:'data.frame':    4 obs. of  16 variables:
#>  $ external: NULL
#>  $ trim    :List of 5
#>   ..$ alg.keep  : chr [1:2] "HC_Euclidean" "KM_Euclidean"
#>   ..$ alg.remove: chr [1:2] "PAM_Euclidean" "DIANA_Euclidean"
#>   ..$ rank.agg  :List of 1
#>   ..$ top.list  :List of 1
#>   ..$ data.new  :List of 1
```

The return value shows which algorithms were kept, removed (if any), and the trimmed (and potentially reweighed) cluster ensemble.

Significance Testing

To test whether there are four statistically distinct clusters (k = 4) versus no clusters (k = 1) using the PAM algorithm, we run `sigclust` with 50 simulations and generate a p-value for this significance test.

```
set.seed(1)
pam_4 <- ccomb_class2$`4`[, "PAM_Euclidean"]
sig_obj <- sigclust(hgsc, k = 4, nsim = 100, labflag = 0, label = pam_4)
co <- capture.output(str(sig_obj))
strwrap(co, width = 80)
#>  [1] "Formal class 'sigclust' [package \"sigclust\"] with 10 slots"
#>  [2] "..@ raw.data : num [1:100, 1:50] -0.0107 -0.7107 0.8815 -1.0851 -0.9322 ..."
#>  [3] ".. ..- attr(*, \"dimnames\")=List of 2"
#>  [4] ".. .. ..$ : chr [1:100] \"TCGA.04.1331_PRO.C5\" \"TCGA.04.1332_MES.C1\""
#>  [5] "\"TCGA.04.1336_DIF.C4\" \"TCGA.04.1337_MES.C1\" ..."
```

```
#>  [6] ".. .. ..$ : chr [1:50] \"ABAT\" \"ABHD2\" \"ACTB\" \"ACTR2\" ..."
#>  [7] "..@ veigval : num [1:50] 11.81 4.51 2.66 2.29 1.84 ..."
#>  [8] "..@ vsimeigval: num [1:50] 11.81 4.51 2.66 2.29 1.84 ..."
#>  [9] "..@ simbackvar: num 0.42"
#> [10] "..@ icovest : num 2"
#> [11] "..@ nsim : num 100"
#> [12] "..@ simcindex : num [1:100] 0.67 0.634 0.641 0.637 0.606 ..."
#> [13] "..@ pval : num 0.14"
#> [14] "..@ pvalnorm : num 0.163"
#> [15] "..@ xcindex : num 0.63"
```

The p-value is 0.14, indicating we do not have sufficient evidence to conclude there are four distinct clusters. Note that we did not use the full `hgsc` data set in this example, so the underlying biological mechanisms may not be fully captured.

References

1. Estivill-Castro, Vladimir (20 June 2002). "Why so many clustering algorithms — A Position Paper". ACM SIGKDD Explorations Newsletter. 4 (1): 65–75.
2. Brendan J. Frey; Delbert Dueck (2007). "Clustering by passing messages between data points". Science. 315 (5814): 972–976.
3. C. Fraley, A. E. Raftery, T. B. Murphy and L. Scrucca (2012). mclust Version 4 for R: Normal Mixture Modeling for Model-Based Clustering, Classification, and Density Estimation. Technical Report No. 597, Department of Statistics, University of Washington.
4. Campello, R. J. G. B.; Moulavi, D.; Sander, J. (2013). Density-Based Clustering Based on Hierarchical Density Estimates. Proceedings of the 17th Pacific-Asia Conference on Knowledge Discovery in Databases, PAKDD 2013, Lecture Notes in Computer Science 7819, p. 160.
5. Campello, Ricardo JGB, et al. "Hierarchical density estimates for data clustering, visualization, and outlier detection." ACM Transactions on Knowledge Discovery from Data (TKDD) 10.1 (2015): 5.
6. G. Govaert and M. Nadif. Latent block model for contingency table. Communications in Statistics - Theory and Methods, 39(3):416–425, 2010.
7. Isa, Dino, V. P. Kallimani, and Lam Hong Lee. "Using the self organizing map for clustering of text documents." Expert Systems with Applications 36.5 (2009): 9584-9591.
8. Andrew Y. Ng, Michael I. Jordan, Yair Weiss On Spectral Clustering: Analysis and an Algorithm Neural Information Processing Symposium 2001
9. Brunet J, Tamayo P, Golub TR and Mesirov JP (2004). "Metagenes and molecular pattern discovery using matrix factorization." Proceedings of the National Academy of Sciences of the United States of America, 101(12), pp. 4164-9.
